# Oral rehabilitation and associated quality of life following mandibular reconstruction with free fibula flap: a cross-sectional study

**DOI:** 10.3389/fonc.2024.1371405

**Published:** 2024-03-18

**Authors:** Lucas M. Ritschl, Hannes Singer, Franz-Carl Clasen, Bernhard Haller, Andreas M. Fichter, Herbert Deppe, Klaus-Dietrich Wolff, Jochen Weitz

**Affiliations:** ^1^ Department of Oral and Maxillofacial Surgery, TUM School of Medicine and Health, Technical University of Munich, Klinikum rechts der Isar, Munich, Germany; ^2^ Institute of AI and Informatics in Medicine, TUM School of Medicine and Health, Technical University of Munich, Klinikum rechts der Isar, Munich, Germany; ^3^ Department of Oral and Maxillofacial Surgery, Josefinum, Augsburg and Private Practice Oral and Maxillofacial Surgery im Pferseepark, Augsburg, Germany

**Keywords:** mandibular reconstruction, free fibula flap, dental implants, oral rehabilitation, quality of life

## Abstract

**Introduction:**

Mandibular reconstruction with the free fibula flap (FFF) has become a standardized procedure. The situation is different with oral rehabilitation, so the purpose of this study was to investigate the frequency of implant placement and prosthetic restoration. Additionally, the patients’ situation, motivation, and treatment course were structurally assessed.

**Materials and methods:**

All cases between January 2013 and December 2018 that underwent mandibular reconstruction in our department with a free fibula flap and gave written informed consent to participate were interviewed with two structured questionnaires about their restoration and quality of life. Additionally, medical records, general information, status of implants and therapy, and metric analyses of the inserted implants were performed.

**Results:**

In total 59 patients were enrolled and analyzed in this monocentric study. Overall, oral rehabilitation was achieved in 23.7% at the time of investigation. In detail, implants were inserted in 37.3% of patients and showed an 83.3% survival of dental implants. Of these implanted patients, dental implants were successfully restored with a prosthetic restoration in 63.6. Within this subgroup, satisfaction with the postoperative aesthetic and functional result was 79.9% and with the oral rehabilitation process was 68.2%. Satisfaction with the implant-borne prosthesis was 87.5%, with non-oral-squamous-cell-carcinoma patients being statistically significantly more content with the handling (*p*=0.046) and care (*p*=0.031) of the prosthesis.

**Discussion:**

Despite the well-reconstructed bony structures, there is a need to increase the effort of achieving oral rehabilitation, especially looking at the patient’s persistent motivation for the procedure.

## 1 Introduction

Mandibular reconstruction with the free fibula flap (FFF) is the gold standard for bridging mandibular continuity defects of nearly any extent and cause, since it was first described by Hidalgo (1, 2), and is nowadays performed highly standardized (3–5). But despite the overall good healing rates of the FFF for mandibular reconstruction, the frequency of dental implant placement is only around 30%, as Brown et al. pointed out in their meta-analysis (6). Moreover, orally rehabilitated patients with sufficient gingiva- or implant-supported restoration or prosthesis range only between 2 and 50% according to the literature (7–10). These low and inconsistent rates are surprising since the FFF has good bone quality with stable bone volume over time (11, 12) with consequently good healing of endosseous implants in the FFF (7, 13, 14). Explanations for this apparent contradiction could be the following points: complex patient population (compliance), the frequent need for adjuvant radiation therapy, a positive radiation history, recurrences in the treatment phase, the challenging intraoral situation and costs for healthcare system and patients (15, 16). Further, the postoperatively changed intraoral anatomical situation can negatively influence a sufficient impression and make a stable, sustainable implant placement impossible in the preliminary stage already (10, 15, 16). There is controversy in the literature regarding the effect of radiation therapy and the timing of implant placement in the FFF. Some studies describe a negative impact of adjuvant radiation on implant survival (13, 17–20).

The purpose of this retrospective conducted cross-sectional study was to determine the rate of oral rehabilitation, patient satisfaction during or after the treatment course, and the associated quality of life.

## 2 Materials and methods

### 2.1 Compliance with ethical standards and patient collective

**Figure 1:**
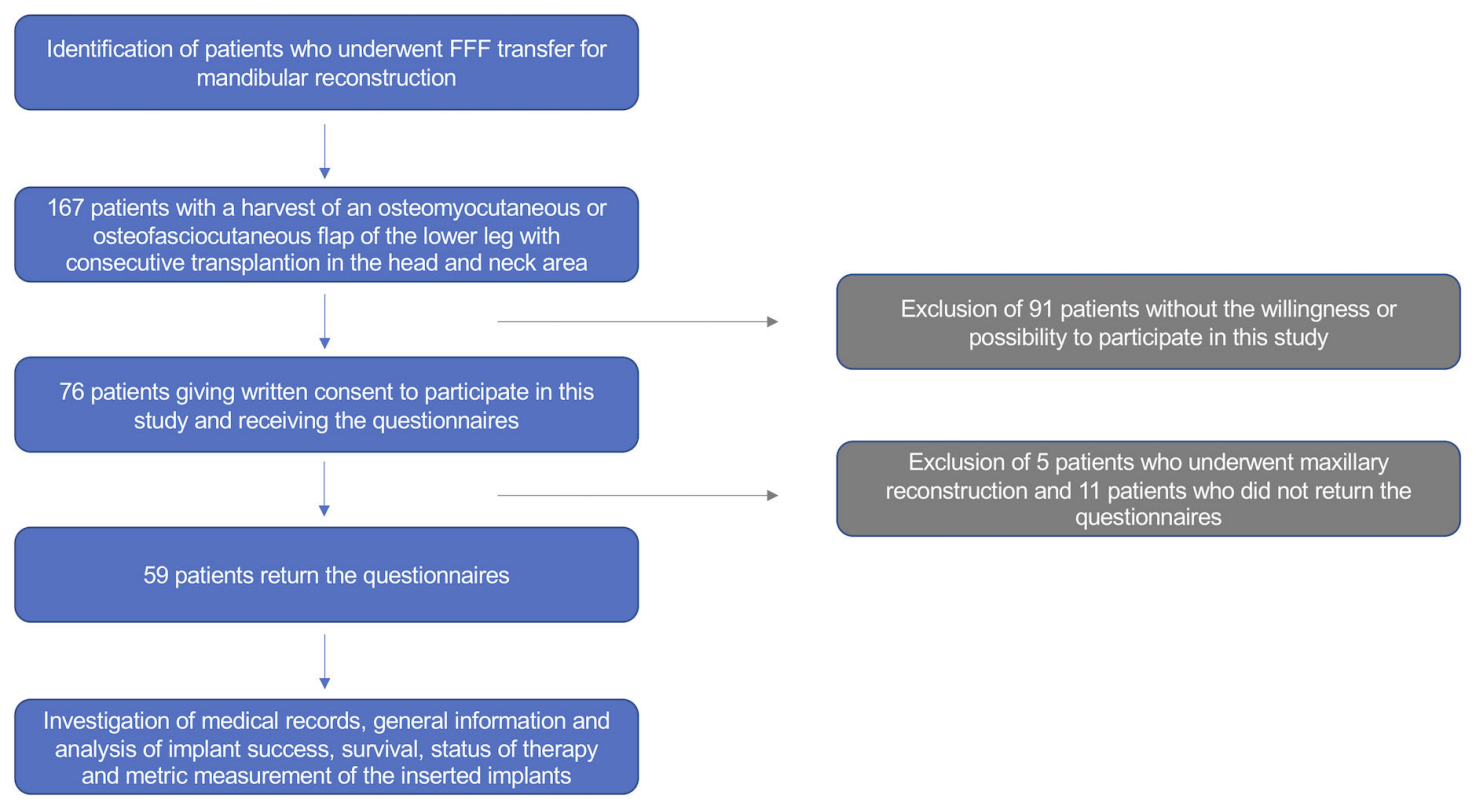
Flowchart of patient enrollment.

All patients undergoing mandibular reconstruction with an FFF between January 2013 and December 2018 were consulted. Of these, only those who agreed to participate (written consent) in the study were included (**Figure 1**). All clinical investigations and procedures were conducted according to the principles expressed in the Declaration of Helsinki. This cross-sectional study was approved by the Ethical Committee of the Technical University of Munich, TUM School of Medicine and Health (Approval No. 459/18S-KK).

Mandibular reconstruction with the FFF was principally performed in a standardized way with regard to surgical approach and raising using cutting guides (either CAD/CAM or ReconGuide) (21). Further, we only use individually preformed 2.0 miniplates for osteosynthesis, which were removed prior to implantation. A volume reduction of the intraoral skin island and vestibuloplasty was performed during plate removal if necessary.

### 2.2 Questionnaires

Patients were concomitantly interviewed with two structured questionnaires, designed for this study in our clinic, which focus mainly on the oral rehabilitation itself and are not yet validated. The first questionnaire captured the current medical and especially dental treatment situation aiming at the oral restoration stage, since patients did not attend the clinic for clinical examination and the potential prosthetic restauration is provided ex house. The second one highlighted social participation, including physical impairment and aesthetic satisfaction (**Table 1**). In addition, the patient’s wishes, treatment course, and satisfaction with the oral rehabilitation were questioned. In accordance with the nature of this cross-sectional study, patients were interviewed and included at different points in their treatment history.

**Table 1:** Questions, their items and metrics regarding the operation and postoperative radiotherapy.

**Questions** **Metrics**	Was there a desire for a prosthetic rehabilitation (1) prior to/(2) after the operation? Was there a desire for a prosthetic rehabilitation (3) prior to/(4) after the radiotherapy?
**100**	Yes, very urgently. The prospect of a prosthesis has increased the interest in the operation for me.
**75**	Yes, mostly. The prosthetic restauration was important to me, but not mandatory.
**50**	In principle, yes, but a prosthesis would not have influenced the decision.
**25**	No, the entire process is too complicated and without clear advantage.
**0**	No, I was always indifferent to a prosthetic restauration or there was no information about a possible prosthetic restauration.

### 2.3 Medical records and analysis of implant-related parameters

In addition, medical records and general information were registered. Further, the time interval for endosseous implant placement [Straumann SLActive standard or standard plus implants (Straumann GmbH; Freiburg, Germany) or Xive implants (Dentsply Sirona Deutschland GmbH; Bensheim, Germany)], and the duration until consecutive prosthetic restoration were recorded. In this way, analyses of implant success, implant survival, and status of oral rehabilitation were recorded. In coordination with the respective dentist, a removable prosthesis on an implant-supported bar construction was chosen (22). In this study, implant quality of health was defined on the basis of the Pisa consensus classification. Accordingly, success required “optimum conditions” namely <2 mm crestal bone loss and the implant being prosthetically loaded. Survival was defined with a crestal bone loss to range from >2 mm to less than half of the implant length. Whereby failure was defined as an implant impossible to load or with crestal bone loss >4 mm and >50% of implant length. Implants that were lost prior to this study (August 2020) were included in the failure group (23).

**Figure 2:**
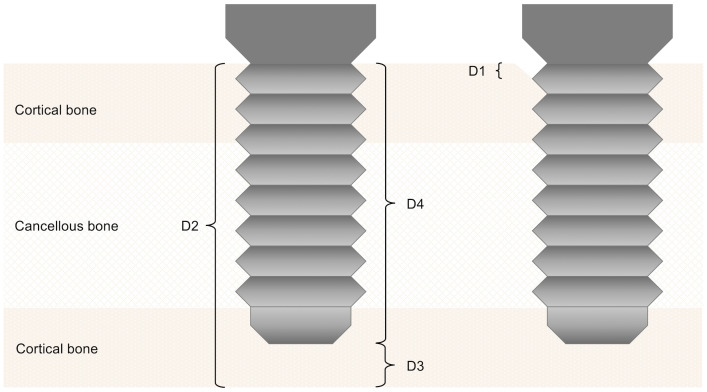
Assessment of peri-implant bone resorption according to Kniha et al. (D 1–4) (23).

Peri-implant bone resorption was assessed according to Kniha et al. at the defined regions of interests D 1–4 using available radiographs (**Figure 2**) (24) and implant-specific complications were also addressed. The gold standard of evaluation, the single-tooth peri-apical radiograph, was mostly not applicable because of the challenging anatomy in the reconstructed oral cavity. This necessitated the use of panoramic radiographs, which were calibrated using the known metrics of the dental implants used. For comparability and equality in the evaluation, we decided to only use panoramic radiographs.

### 2.4 Statistical analysis

For categorical data absolute and relative frequencies are shown, and quantitative data are described by mean and standard deviation or median and interquartile range [1^st^ to 3^rd^ quartile]. For the analysis of pre- and postoperative differences in patient assessments, the Wilcoxon signed-rank test was used. For the assessment of mean differences between relevant groups, linear regression models were fitted to the data. All statistical tests were performed on an exploratory two-sided 5% significance level. No adjustments were made for multiple testing. For relevant effect sizes, 95% -confidence intervals are presented. Analysis was done with IBM SPSS 27 for Mac software (IBM Corp, Armonk; New York, United States).

## 3 Results

### 3.1 Patient collective, mandibular reconstruction, and medical records

The final population consisted of 59 patients, excluding those with maxillary reconstruction or who did not return the questionnaires. The gender distribution, age, and operation as well as hospitalization data of the enrolled patients are shown in **Table 2**.

**Table 2:** Overview of enrolled patients with regard to registered parameters: gender, age, indication for surgery, mandibular defect class according to Brown et al. (25), number of segments.

Parameters			
**Gender female/male**	20 (33.9%)/39 (66.1%)		
**Age median [years] (range)**	60 (17–82)		
**Indication**	OSCC ORN MRONJ Osteomyelitis Aneurysmatic bone cyst Ameloblastoma Keratocyst		27 (45.8%) 17 (28.8%) 6 (10.2%) 6 (10.2%) 1 (1.7%) 1 (1.7%) 1 (1.7%)
			Operation time in minutes (median and 1^st^; 3^rd^ quartile)
**Number of segments and operation time [minutes]**	1 2 3	38 (64.4%) 10 (16.9%) 11 (18.6%)	554 [480; 613] 613 [522; 655] 576 [524; 694]
			Hospital stay in days (median and 1^st^; 3^rd^ quartile)
**Number of segments and hospital stay [days]**	1 2 3	38 (64.4%) 10 (16.9%) 11 (18.6%)	16 [12; 22] 16 [14; 31] 19 [12.5; 25]

OSCC, oral squamous cell carcinoma; ORN, osteoradionecrosis; MRONJ, medication-related osteonecrosis of the jaw.

### 3.2 Questionnaires

Patients were interviewed with two structured questionnaires, as described in section 3.2. The results of the first questionnaire are following coherently as a part of section 3.7, stating the oral rehabilitation status. The second questionnaire had 5 options to answer per question, punctuating 0–4 points and ascending from the worst to the best possible answer. Questions 4 and 5 asked how confident patients were in familiar and unfamiliar environments. The possible answers aimed at the physical impairment after FFF raising, including lack of proprioception and mobility. Question number 6 sought to determine how satisfied patients were with their appearance and if it impairs their choice of acquaintances or localities they see and go to. The results were generally satisfying since the mode was the best answer to all three questions. While Questions 4 and 6, including moving in familiar environments and the satisfaction of appearance, were only answered with the worst option in 5.3 and 5.5% respectively, question 5, with moving in unfamiliar environments, was answered in 17,5% with the worst option (**Table 3**).

**Table 3:** Results of questionnaire regarding the patient´s wish for oral rehabilitation at different time points of treatment timeline.

Metrics	pre-OP	post-OP	*p*-value	pre-RTx	post-RTx	*p*-value
**100**	25 (47.2%)	33 (60%)	0.013	5 (31.3%)	5 (33.3%)	0.414
**75**	6 (11.3%)	5 (9.1%)		5 (31.3%)	3 (20%)	
**50**	10 (18.9%)	11 (20%)		4 (25%)	4 (26.7%)	
**25**	2 (3.8%)	3 (5.5%)		0 (0%)	2 (13.3%)	
**0**	10 (18.9%)	3 (5.5%)		2 (12.3%)	1 (6.7%)	

pre-OP, preoperative; post-OP, postoperative; pre-RTx, prior to radiation therapy; post-RTx, after radiation therapy.

The desire for a prosthetic reconstruction, the query shown in **Tables 1**, **3**, increased significantly in the course of the treatment in a comparison of the pre- and postoperative motivation in our questionnaires (*p*=0.013). The motivation for oral rehabilitation before and after radiotherapy showed no significant difference (*p*=0.444) but pointed to an increase. Generally, the planning was welcomed by the patients, although the interval between the mandibular reconstruction and the start of the planning, as well as its duration, were often criticized. Additionally, patients with malignancies (and consecutive adjuvant radiotherapy) were significantly less satisfied with the handling (mean difference of 30 points, 95% confidence interval 6 to 60 points, *p*=0.046) and care (33 points, 95% ci 3 to 63 points, *p*=0.031) of the prosthesis.

### 3.3 Incidence of dental implant insertion and oral rehabilitation

Dental implant insertion was accomplished in accumulative 22 patients (37.3%) at the time point of analysis, with 15 being implanted in our institution and 7 elsewhere. The median time from reconstructive surgery to dental implantation was 366 days (0–1,262). Completed oral rehabilitation was reported in 63.6% (14/22) of the implanted cases and overall 23.7% (14/59) of enrolled patients at the time of data collection in this cross-sectional study. The median time from reconstructive surgery to oral rehabilitation with a removable prosthesis on an implant-supported bar construction was 750 days (224–1,447). After eight implant losses, a total of 52 dental implants (median 4; range 1–6) in 15 patients were evaluated radiographically. There was no data available for seven out of 22 patients (see below). The incidence of dental implant insertion and oral rehabilitation in context with gender, age, and clinical data is illustrated in **Table 4**.

**Table 4:** Incidence of dental implant insertion and oral rehabilitation of the enrolled 59 patients, of whom 22 received dental implants at the time of data collection in this cross-sectional study with regard to different potentially confounding parameters.

Parameters		Implantation y/t	Oral rehabilitation y/t
**Gender**	Female (33.9%) Male (66.1%)	6/20 16/39	6/20 8/39
**Age [years]**	≤19 (1.7%) 20–39 (1.7%) 40–59 (47.5%) 60–79 (47.5%) ≥80 (1.7%)	1/1 1/1 12/28 11/28 1/1	1/1 1/1 4/28 7/28 1/1
**Indication**	Benign Malign	13/32 9/27	11/32 3/27
**Number of segments**	1 (64.4%) 2 (16.9%) 3 (18.6%)	13/38 1/10 3/11	10/38 0/10 4/11
**Type of FFF**	Myo-osseous Osteomyocutaneous	8/15 14/44	7/15 7/44
**Adjuvant RTx**	Yes No	7/19 15/40	2/19 12/40

y/t, yes/total; FFF, free fibula flap; RTx, radiation therapy.

### 3.4 Evaluation of implants

There were 60 implants placed in 15 patients. The other seven out of 22 patients were implanted elsewhere and/or without radiography and consequently without an implant count and evaluation in this chapter. The median duration of surveillance was 32 months (15–56). A total of eight implant losses occurred in three patients, with one patient losing all three implants. So, 52 out of 60 (88.6%) implants were still *in situ* in August 2020.

We defined survival and success of implants one the basis of the 2008 Pisa consensus in four groups as follows: I (= success), II (= satisfactory survival), III (= compromised survival), and IV (failure) (23). Forty-two implants were classified in group I, seven in group II, one in group III, and two in group IV. Considering that an implant should be loaded and in group I to be completely successful, a total of 28 out of 52 (53.8%) matched those criteria. Including those, that makes a total of 50 out of 60 (83.3%) implants surviving, when looking at the implants only and not considering the loading of them with prosthetics.

The radiographs of the 15 patients with 52 surviving dental implants were assessed according to Kniha et al. (24). Due to incomplete radiography 52 implants and 104 crestal bone levels (D 1) were evaluated in total. Crestal bone loss (D1) was <2 mm in 83, 2–4 mm in 16, and >4 mm in five measurements. Looking at D1 mesial and distal of single implants, 37 of them showed a crestal bone loss <2 mm, 23 ranged from 2–4 mm, and two cases had a bone loss of >4 mm.

**Table 5** shows the results of the binary logistic regression analyses of potential confounding factors (age, gender, BMI, indication, flap type, number of FFF segments and adjuvant radiotherapy) on implant survival and incidence of oral rehabilitation.

**Table 5:** Binary logistic regression analyses of potential confounding factors on implant survival and incidence of oral rehabilitation.

Parameter	Implant survival	Oral rehabilitation
	*p*-value (95-% confidence interval)	*p*-value (95-% confidence interval)
Age	0.553 (0.864 – 1.081)	0.814 (0.939 – 1.051)
Gender	0.598 (0.111 – 45.723)	0.107 (0.015 – 1.510)
BMI	0.293 (0.768 – 2.396)	0.886 (0.715 – 1.099)
Indication	0.764 (0.570 – 2.153)	0.111 (0.884 – 3.298)
Flap type	0.758 (0.081 – 31.771)	0.146 (0.039 – 1.623)
Number of FFF segments	0.666 (0.150 – 3.364)	0.399 (0.287 – 1.644)
Adjuvant RTx	0.999 (0.000)	0.071 (0.024 – 1.164)

BMI, body mass index; FFF, free fibula flap; RTx, radiation therapy.

## 4 Discussion

### 4.1 Clinical situation and patient collective

The fibular bone quality is reported as good and reliable for dental implant insertion and the FFF transfer for mandibular reconstruction has reached meanwhile a highly standardized level. This leads consequently to an increasing number of studies thematizing oral rehabilitation. But this final step is because of diverse reasons surgically demanding in a heterogenous and challenging cohort. An implant-borne prosthesis is in most cases a favorable solution for oral rehabilitation due to the complex anatomy and function of the oral cavity following ablative surgery and bony reconstruction (26). Yet it remains more problematic in the management of the soft tissue since the FFF is often necessary in tumor cases, and therefore a skin island is also required. This is often too voluminous, remains mobile and levels the vestibulum. Ultimately, at least one pre-implantological correction of this unfortunate situation is required (volume reduction and vestibuloplasty) to reduce the secondary occurrence of mucosal or osseous peri-implantitis (27). This situation requires a high level of patient compliance and surgical expertise, as the soft tissues may react differently due to perceived irradiation in the case of malignancies, and one risks a wound-healing disorder with secondary loss of the vascularized bone transplant.

The literature on this topic is consecutively very heterogeneous (in terms of indication, positive radiation history, nicotine abuse, extent of resection, number of bone segments, etc.) and also uses different definitions for survival and success. For this reason, we have opted for the Pisa consensus definition in order to enable a certain degree of comparability in our cross-sectional study, which analyzed 59 patients with a female/male 33.9%/66.1% distribution. This is comparable to a systematic review by Wijbenga et al. (28). But in terms of age, Wijbenga et al. describe a mean of 50.9 years, which is lower than the 60.6 years mean in our study (28). The indication for the bony reconstruction was similar as described by Hundepool et al., with the largest proportion of patients suffering from oral squamous cell carcinoma (45.8% vs. 63%) and the second largest suffering from osteoradionecrosis (28.8% vs. 20%) (29).

### 4.2 Questionnaires – quality of life

Since we designed the questionnaires, the comparability to other literature is limited. We defined the quality of life with the satisfaction with their appearance as well as the feasibility and confidence in participation in daily life with different settings and companions. Schliephake and Jamil pointed out that oncologic surgery for oral cancer led to a “significant decrease in oral function with reduced body image and reduced ability and willingness for social contact” (30). This is endorsed by Löfstrand et al., who described a significantly worsened social functioning and role functioning in the SF-36 compared to reference populations (31). Besides the psychosocial impairment physical problems like ankle instability, weakness, toe contraction and decreased range of motion are occurring (32). Our study suggests increased satisfaction with those parameters. It needs to be pointed out that the oncologic surgery took place years before our questionnaires and study, and so patients have already adapted and accepted the new situation. On the one hand this fact might lead to better results in this questionnaire regarding the will and interest for oral rehabilitation, because the negative memories of the exhausting and debilitating ablative and reconstructive surgery with adjuvant therapy have faded over time. At the time of the study, there was a significant increase in the desire for oral rehabilitation in the included population. On the other hand, it could have been that there was no interest in further surgical interventions that are necessary due to dental implantation (vestibuloplasty, removal of the osteosynthesis plates, implantation and secondary exposure, etc.), the associated surgical risks including loss of the FFF and further hospitalization. Patients with a bony reconstruction of the mandible are scarce. This could lead to insufficient prosthodontic treatment by the private dental practitioner. Fierz et al. pointed out that prosthodontic treatment in tumor patients is close interdisciplinary cooperation, with 13 appointments common (26). The significance of oral rehabilitation is underlined by Hundepool et al., who found statistical significance when it came to social participation in the H&N35 pre- and post-oral rehabilitation (29). Although there is no significant difference in quality of life in terms of flap type in the literature, there is a tendency toward the better in patients with FFF, according to Moubayed et al. (33).

### 4.3 Dental implant insertion, survival, success, and oral rehabilitation

The interval of secondary dental implantation in FFF is in line with the literature in our study (mean time of 12.9 months). Some others reported a shorter interval with 9.4 (34), and others had a longer interval with 17.5 months (29) until dental implant placement. Also, the duration of treatment from bony reconstruction to oral rehabilitation was nearly similar at 23.9 months compared to the 22.4 months described by Parbo et al. (34).

Survival rates reported in the literature range from 87–97% in five- to ten-year surveillance (19, 35, 36). In our cross-sectional study, the survival rate of implants in the median duration of surveillance of 32 months (15–56) was 83.3% which is lower. This could be the result of an older cohort as stated above, although Sendyk et al. meta-analyzed that age is not a risk factor for dental implant loss, at least in the original bone (37). As reported by others, radiotherapy did not negatively influence the success rate (38).

Implant success is defined very heterogeneously in the literature and there is yet to find a gold-standard (39). We defined the success of implants as the implant being *in situ* and loaded, and furthermore with a bone loss <2 mm, but not exceeding half of the length of the dental implant, according to the 2008 Pisa consensus definition (23). It needs to be underlined that the cohort in this study was known to be challenging in terms of oral rehabilitation. The lower implant success in this study compared to others is mainly due to a lack of oral rehabilitation, which was partly in progress in some enrolled participants. Having this in mind, the duration of surveillance was shorter than in comparable studies like Hundepool et al., which is a cause of the lower prosthetic restoration lowering the success rate (29). Besides that, the duration of treatment from bony reconstruction to oral rehabilitation was nearly similar at 23.9 months compared to 22.4 months described by Parbo et al. (34).

We did not collect the past and current status of smoking and oral hygiene. So apart from the missing clinical examination, the lack of information impedes the determination of possible reasons for the impaired implant survival. However, most implants that were surveilled and did not fail were healing properly, since only two out of 52 did not match the 2008 Pisa criteria (23). Pellegrino et al. described an overall implant success of 95.4% at 12-month follow-up and 73.5% at 60-month follow-up (14), which is an indicator, that long-term success in implant placement in FFF is challenging. In our study, the timing with regard to primary or secondary implantation showed no difference in healing and success. This differs from Panchal et al., who reported in a meta-analysis about implant survival in vascularized bone flaps an estimated implant success rate of 97% in implants placed immediately and 89.9% in delayed implant placement, with a mean follow-up ranging from 14 to 40 months. Also, Hessling et al. noted that implantation in hard tissue reconstruction significantly decreased implant survival compared to the mandible, with the FFF being, in their study, statistically significantly more likely to fail compared to other bone grafts for dental implants (40).

### 4.4 Limitations

Some limitations of this study include the heterogeneity of the cohort regarding indication and a retrospective analysis of data without clinical examination. The implant survival rate may therefore be overestimated since clinical signs of failure were not evaluated. Furthermore, data is derived exclusively from the questionnaires. Patients could have misunderstood the questions or just did not know what their private dental practitioner is planning. Another problem is the use of panoramic radiographs instead of single-tooth peri-apical radiographs, which is due to the anatomical obstacles after reconstructive surgery. Also, there is no structured radiographic recall protocol that defines marks of when X-ray has been used to inspect the implant. Furthermore, there is a German bureaucracy and administration issue as there exist two types of insurance: public and private insurance (41). This leads in our case to an inadequacy of treatment, as the costs of privately insured patients are usually covered by the insurance provider more quickly. In publicly insured patients, the costs are not always covered and need to be applied for by the surgeon and the dentist beforehand. An application for an exemption must be made in accordance with §28 of the Social Insurance Act V for the covering of costs associated with oral rehabilitation by the insurance. We therefore believe that the direct costs are not necessarily the reason for such a low rate in Germany, but rather the associated bureaucratic and time-consuming effort, as it is not uncommon for expert opinions to have to be prepared by third parties. Although the outcome is physically not impaired (42), the results of our survey indicate a significantly rising will for prosthesis after surgery, therefore resulting in a delay in therapy. In conclusion, the complete oral rehabilitation is mostly just being delayed by this administrative process. Although it does not impair the outcome of the implants, patients are being led down a prolonged path of therapy toward oral rehabilitation, which negatively affects their quality of life. Nowadays bony reconstruction is mostly CAD/CAM planned. The ideal position of the neomandibula (in terms of shape of the original mandible, relation to the maxilla, and crestal bone height for implant placement) is therefore predestined and mostly achieved (43). This is underlined by Tran et al., who stated that with in-house virtual surgical planning FFF were more amenable to dental implants as compared to freehand surgery (44).

### 4.5 Interpretation

Mandibular reconstruction is today a very standardized procedure with good postoperative results regarding symmetry and accuracy. Based on the increased use of CAD/CAM technology and the preoperative planning of a close-to-perfect reconstruction of the tooth-bearing mandibula, the chances of improvements in oral rehabilitation can cautiously be seen positively. Within the limits highlighted in this study, the reconstructive surgeon can achieve a good base for oral rehabilitation when using the free fibula flap, since patient motivation for a prosthesis is increasing significantly. Furthermore, non-OSCC patients are significantly more content with their implant-borne prosthesis. An interdisciplinary approach to ensure a satisfactory process and outcome of oral rehabilitation is necessary. Good guidance for patients after a long therapy path should be the goal of the physician.

## Data availability statement

The raw data supporting the conclusions of this article will be made available by the authors, without undue reservation.

## Ethics statement

The studies involving humans were approved by Ethical Committee of the Technical University of Munich, School of Medicine and Health (Approval No. 459/18S-KK). The studies were conducted in accordance with the local legislation and institutional requirements. Written informed consent for participation was not required from the participants or the participants’ legal guardians/next of kin in accordance with the national legislation and institutional requirements. The animal study was approved by Ethical Committee of the Technical University of Munich, TUM School of Medicine and Health (Approval No. 459/18S-KK). The study was conducted in accordance with the local legislation and institutional requirements.

## Author contributions

LR: Writing – original draft, Visualization, Supervision, Formal analysis, Conceptualization. HS: Writing – original draft, Supervision, Software, Investigation, Formal analysis. F-CC: Writing – review & editing, Software, Investigation, Formal analysis, Data curation. BH: Writing – review & editing, Methodology, Formal analysis, Data curation. AF: Writing – review & editing, Visualization, Validation, Supervision, Software. HD: Writing – review & editing, Validation, Supervision, Conceptualization. K-DW: Writing – review & editing, Supervision, Resources, Project administration, Conceptualization. JW: Writing – original draft, Supervision, Project administration, Conceptualization.
